# Stereotactic body radiotherapy for spine and non-spine bone metastases in prostate carcinoma – a multicenter cohort analysis

**DOI:** 10.1016/j.jbo.2025.100710

**Published:** 2025-09-11

**Authors:** Franziska Nägler, Isabell Seiler, Sebastian Schäfer, Johannes Meents, Fabian Lohaus, Arne Grün, Olaf Wittenstein, Kenneth Klischies, Julia Remmele, Alexander Rühle, Miriam Eckl, Oliver Blanck, Judit Boda-Heggemann, Frank A. Giordano, Christos Moustakis, Nils H. Nicolay, Lena Kästner

**Affiliations:** a Department of Radiotherapy and Radiation Oncology, University Hospital Leipzig, Stephanstraße 9a, Leipzig, Germany; b Comprehensive Cancer Center Central Germany, Partner Site Leipzig, Leipzig, Germany; c Department of Radiation Oncology, University Medicine Mannheim, Medical Faculty Mannheim, Heidelberg University, Theodor-Kutzer-Ufer 1-3, Mannheim, Germany; d DKFZ Hector Cancer Institute at the University Medical Center Mannheim, Mannheim, Germany; e Mannheim Institute for Intelligent Systems in Medicine (MIISM), Medical Faculty Mannheim, Heidelberg University, Germany; f National Center for Tumor Diseases (NCT), NCT/UCC Dresden, a partnership between DKFZ, Faculty of Medicine and University Hospital Carl Gustav Carus, TUD Dresden University of Technology, and Helmholtz-Zentrum Dresden-Rossendorf (HZDR), Germany; g OncoRay – National Center for Radiation Research in Oncology, Faculty of Medicine and University Hospital Carl Gustav Carus, TUD Dresden University of Technology, Helmholtz-Zentrum Dresden-Rossendorf, Dresden, Germany; h German Cancer Consortium (DKTK), Partner Site Dresden, and German Cancer Research Center (DKFZ), Heidelberg, Germany; i Department for Radiation Oncology, Charité – Universitaetsmedizin Berlin, corporate member of Freie Universitaet Berlin, Humboldt-Universitaet zu Berlin, and Berlin Institute of Health, Germany; j Department of Radiotherapy and Radiation Oncology, University Hospital Schleswig-Holstein, Kiel, Germany

**Keywords:** Bone metastases, Prostate cancer, Radiotherapy, Stereotactic radiotherapy, Metastasis-directed therapy (MDT), Spine metastases, Non-spine metastases

## Abstract

**Background and purpose:**

Metastases-directed radiotherapy plays an increasing role in oligometastatic prostate cancers (OMPC). Here, we investigated the role of stereotactic body radiotherapy (SBRT) for spine and non-spine bone metastases (BoM) from prostate cancer in a large real-world multicenter cohort.

**Material and methods:**

This multicenter cohort analysis from five tertiary cancer centers included patient data of spine and non-spine BoM irradiated between 2010 and 2024. Overall survival (OS), progression-free survival (PFS), local recurrence-free survival (LRFS), SBRT target volumes and doses, toxicity, and the role of additional systemic therapies were evaluated retrospectively.

**Results:**

231 patients (341 BoM) with median follow-up time of 28.3 months were included. Most common localization were spine (39.3 %), pelvic bone (31.7 %), and ribs (17.9 %). 1- and 5-year PFS for spine BoM were 93.8 % (95 %CI:84.2–97.6 %) and 32.1 % (95 %CI:16.8–44.4 %) and for non-spine BoM 91.7 % (95 %CI:85.1–95.5 %) and 36.6 % (95 %CI:25.8–47.5 %), respectively. 1- and 5-year OS for spine BoM amounted to 94.2 % (95 %CI:85.3–97.8 %) and 69.2 % (95 %CI:50.2–82.2 %) and for non-spine 100 % and 73.3 % (95 %CI:59.1–83.3 %). Older age (p < 0.005) and additional systemic therapies (p = 0.05) were associated with worse OS, older age and larger treatment volumes with worse PFS (p = 0.04). Toxicities were low, with fracture rates of 0.3 % (acute) and 1.2 % (late).

**Conclusion:**

Bone SBRT for OMPC is an effective treatment with low toxicity and particularly low fracture rates for both spine and non-spine BoM with no difference in outcome based on the localization. Prospective trials will help to identify the patients benefitting most from this approach and to establish standardized SBRT concepts incorporating systemic treatments.

## Highlights

• Metastases-directed therapy plays an increasing role in oligometastatic prostate cancer.
• Bone SBRT is an effective treatment with excellent outcome and low toxicity.
• Especially low fracture rates for both spine and non-spine BoM (1.5 %).
• No significant difference in outcome based on localization in spine or non-spine.

## Graphical abstract

Stereotactic body radiotherapy for spine and none-spine bone metastases in prostate carcinoma – a multicenter analysis.

**Figure:**
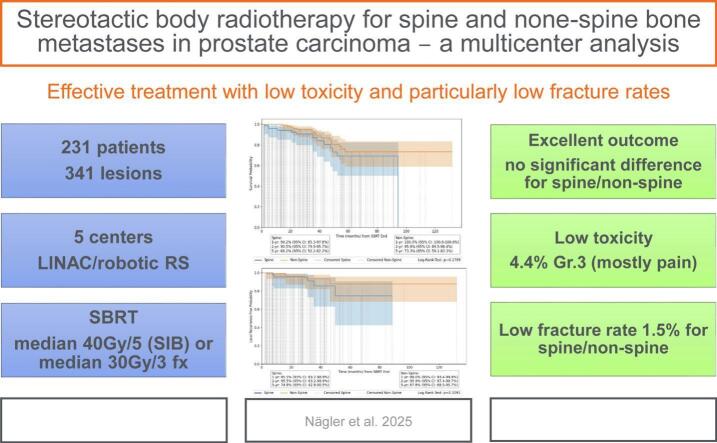

## 1 Background

Prostate cancer is the second most frequent cancer among men worldwide [1]. The number of patients with metastatic disease at time of diagnosis increases due to screening as well as improvements in imaging modalities [2]. Metastases in patients with advanced prostate cancer mainly occur in regional lymph nodes, but also in distant organs, most commonly in bones (70 % of patients) with a majority presenting as osteoblastic lesions [3]. The early detection of limited bone metastases (BoM) is increasing due to steady improvements and broader availability of imaging modalities, such as prostate-specific membrane antigen positron emission tomography/ computed tomography (PSMA-PET/CT) [4,5]. This allows the detection and treatment of oligometastatic stages of prostate cancer (OMPC). Anti-androgen therapy is the standard treatment of advanced prostate cancer, but depending on size and localization, BoM induce a high risk for skeletal-related events (SRE) with approximately one third of BoM considered complicated lesions, leading to pain, pathological fractures (42.1 %) and neurological compromise (36.3 %) [6]. Currently, palliative external beam radiotherapy is a standard treatment for symptomatic BoM, providing successful pain relief with minimal toxicity [7,8]. With improving diagnostic options leading to detection of asymptomatic metastases in early stages of cancer and increasing long-term survival due to new systemic therapies, high-dose ablative radiotherapy becomes increasingly important. It offers the chance to improve long-term tumor and symptom control, especially in the context of oligometastatic disease (OMD). In recent years, several trials have demonstrated the clinical benefit regarding progression-free survival (PFS) and overall survival (OS) [[9], [10], [11], [12], [13], [14]]. In the context of bone OMD, guidelines for bone stereotactic body radiotherapy (SBRT) of spine and non-spine BoM were published in recent years, but important questions regarding the optimal choice between locally ablative and palliative bone SBRT, treatment volumes, optimal dose, as well as patient selection remain unanswered [[15], [16], [17], [18], [19], [20]]. For patients with BoM of OMPC, the long-term analysis of the STOMP/ORIOLE trials suggests a clinical benefit of MDT over observation [21]. A large *meta*-analysis concluded that there were promising improvements in PFS for different OMPC states without excessive toxicities, but OS comparisons were still immature, and the level of evidence was low or moderate [22]. Furthermore, the combination of SBRT with androgen deprivation therapy (ADT) was found to be more effective than ADT (EXTEND trial) or SBRT (RADIOSA trial) alone [23,24].

In order to identify patients who will benefit most from bone SBRT and to examine common SBRT target/ dose concepts and their efficacy and safety, especially regarding the anatomical localization of BoM (spine and non-spine), we pooled and analyzed patient and treatment data from five academic tertiary cancer centers in Germany.

## 2 Patients and methods

### 2.1 Patient selection

Individual clinical and treatment data of patients with BoM from histologically confirmed prostate carcinoma irradiated with bone SBRT between 2010 and 2024 at five academic tertiary cancer centers in Germany were collected and analyzed retrospectively. The diagnosis of BoM was based on radiologic findings in CT, magnetic resonance imaging (MRI), PSMA PET/CT or scintigraphy for patients with histologically confirmed prostate cancer. OMD was classified and then further subclassified according to established European consensus criteria [25]. SBRT characteristics consisting of patient positioning, dose concepts and plan parameters were collected. If applicable, pre-treatment symptoms, additional systemic therapies and necessary salvage therapies after SBRT were recorded and analyzed. Biologically effective dose (BED) was calculated with the formula BED = n × d×(1 + d/(α/β)) with n = number of fractions, d = dose per fraction, and α/β = 4 Gy (BED_4_) for prostate cancer and additionally with the commonly used α/β = 10 Gy (BED_10_) [26]. The minimal dose of SBRT was requested as BED_10_ of at least 45 Gy for the target volume in a maximum of 10 fractions, including concepts with ≥ 1x 18 Gy, ≥3x 8 Gy, ≥5x 7 Gy or similar to the surrounding isodose, as well as simultaneous integrated boost concepts with either 5 or 10 fractions. Thus, palliative dose concepts, such as 5 times 4 Gy or 10 times 3 Gy were excluded.

This study has been approved before initiation by the institutional ethics board (127/24-ek) and was part of an explorating work in preparation of a large project of the national working groups radiosurgery and stereotactic radiotherapy of the German Society for Radiation Oncology (DEGRO) and the German Society of Medical Physics (DGMP). The study followed the STROBE guidelines for reporting observational studies (Supplementary Data 2) [27].

### 2.2 Response assessment

The findings of routinely administered pre- and post-SBRT imaging examinations (including CT, MRI and PET scans) were analyzed regarding response, local recurrence and fractures. Local recurrence was defined as a radiological confirmed progression. If several BoM were treated in a patient, each region was analyzed separately. For calculating local recurrence-free survival (LRFS) after SBRT, lesions were censored at the time of their last imaging. Since imaging is not routinely performed during follow-up of OMPC in the absence of increasing PSA, we additionally assessed rising PSA values post SBRT to calculate biochemical recurrence-free survival (BRFS). OS was calculated from the end of SBRT until death of any cause, and PFS was defined from the end of SBRT until death or any type of progression of the underlying tumor disease. Acute (up to 90 days post SBRT) and late toxicities were documented according to Common Terminology Criteria for Adverse Events (CTCAE) version 5.0 [28].

### 2.3 Statistical analysis

All analyses were implemented using custom Python-based scripting (v3.12.7). Data management and pre-processing were conducted using pandas (v2.2.3) and numpy (v2.2.1), while statistical computations and survival modelling were carried out with scipy (v1.14.1) and lifelines (v0.30.0). Data were visualized using matplotlib (v3.10.0) and seaborn (v0.13.2).

Descriptive analyses of patient demographics and clinical characteristics were performed. Survival analyses employed both Kaplan–Meier estimation and multivariate Cox proportional hazards models. The Cox model incorporated patient age, anatomical site (spine vs. non-spine), mean BED_4_ to the gross tumor volume (GTV), tumor volume, and concomitant/ sequentially systemic therapy (ADT or cytotoxic chemotherapy, details see Table 2) as covariates. Multivariate analyses were performed only on complete cases. Model assumptions were verified using a significance threshold of 0.05. Kaplan–Meier curves were generated to compare survival outcomes across various subgroups and statistically assessed via log-rank tests. Survival probabilities at predefined time points were interpolated and reported with 95 % confidence intervals (95 %CI). We assessed the statistical correlation between BED_4_ and BED_10_ and patient survival probability in 5 Gy increments, beginning at 100 Gy, separately for spinal and non-spinal bone metastases.

**Table 1:** Patient characteristics.

Variable	
*Patients (n = total)*	231
*Bone metastases (n = total)*	341

*Age*	
Median in years (95 %CI)	72 (57–81)

*Karnofsky-Index before SBRT*	
Median in % (95 %CI)	100 (80–100)

*Initial therapy primary tumor*	
Surgery	197 (57.8 %)
Radiotherapy	90 (26.4 %)
Chemotherapy	8 (2.4 %)
Androgen Deprivation Therapy (ADT)	41 (12.0 %)

*Primary tumor/metastases curative treated before SBRT:*	
Yes	68 (29.4 %)
No	146 (63.2 %)
Synchronous treatment	41 (19.2 %)
Metachronous treatment	176 (82.2 %)

*Bone metastases*	
Time between initial diagnosis of prostate cancer and bone metastasis, median (months)	28.3
Time between diagnosis of bone metastasis and start SBRT treatment, median (months)	2.0
Synchronous	41
Metachronous	176
*Classification according ESTRO/ EORTC* [25]	
Metachronous oligoprogression	101 (29.6 %)
Metachronous oligorecurrence	98 (28.7 %)
Repeat oligoprogression	33 (9.7 %)
Repeat oligorecurrence	26 (7.6 %)
Induced oligoprogression	6 (1.8 %)
Induced oligorecurrence	(0.3 %)

*Localization of bone metastases*	
Spine incl. sacrum	136 (39.8 %)
Non-spine	205 (60.2 %)

*Characteristics of bone metastases*	
Radiological osteolytic/ osteoblastic	65 (19.8 %)/ 252 (76.6 %)
Soft tissue infiltration no/ yes	321 (97.6 %)/ 8 (2.4 %)
Symptomatic/ asymptomatic	28 (9.3 %)/ 300 (88.0 %)
Pain before SBRT	24 (7.0 %)

*PSA before SBRT* in ng/ml (95 %CI)	29.2 ng/ml (21.7–36.7)

Abbreviations: CI: Confidence Interval, SBRT: Stereotactic Body Radiotherapy, PSA: Prostate-Specific Antigen.

**Table 2:** Treatment parameters.

Variable	N
*Imaging before treatment planning:*	341 (100 %)
Diagnostic CT	64 (18.8 %)
MRI	48 (14.1 %)
PET (including PSMA-PET)	278 (81.5 %)
Scintigraphy	56 (16.4 %)
*Number of treated bone metastases per patient,* median (range)	1 (1–6)

*Treatment technique*	
LINAC Photon FF/ FFF	108 (32.8 %)/ 186 (56.5 %)
Robotic radiosurgery (Cyberknife)	32 (9.4 %)

*Systemic therapy*	
Chemotherapy (concomitant/ sequentially)	4 (1.2 %)
Antihormonal (concomitant/ sequentially)	210 (61.6 %)
No information available	127 (37.2 %)

Abbreviations: CT: Computed Tomography, MRI: Magnetic Resonance Imaging, PET: Positron Emission Tomography, PSMA: Prostate-Specific Membrane Antigen, LINAC: Linear Accelerator, FF(F): Flattening-Filter(−Free).

## 3 Results

### 3.1 Patient characteristics

Data of 231 patients with 341 stereotactically irradiated BoM from OMPC were analyzed (Table 1) retrospectively. Median age at SBRT was 72 years (95 %CI: 57–81) with a median Karnofsky performance score of 100 % (95 %CI: 80–100). The median number of treated lesions was 1 (range: 1–6) per patient. Primaries were mostly treated with surgery (197/341 lesions; 57.8 %) or radiotherapy (90/341 lesions; 26.4 %). At the time of SBRT of BoM, 61.6 % (210/341) of lesions also received antihormonal therapy (such as Bicalutamide, Leuprorelin, Apalutamide) and 1.2 % (4/341) were treated with chemotherapy (Docetaxel). For 37.2 % (127/341) of lesions, no information about systemic therapies was available. Median time between first diagnosis of prostate cancer and SBRT of BoM was 28.3 months and median time between diagnosis of BoM and start of SBRT was 2 months. BoM were synchronous in 41 cases and metachronous in 176 patients. The most common localization for BoM in this study was found to be the spine (39.8 %), pelvic bones (31.7 %), ribs (17.9 %), and scapula/shoulder (5.9 %). BoM symptoms in our patient cohort were rare with 7 % of patients reporting pain and 7.3 % presenting with fractured bones. Details are outlined in Supplementary Fig. 1.

### 3.2 Treatment parameters/ SBRT patterns of care

Radiotherapy planning was based on a planning CT in treatment position and image fusion with diagnostic imaging. Patients were positioned according to the institutional standards of each center based on national recommendations set up by the working groups of DEGRO and DGMP [29,30].

**Fig. 1:**
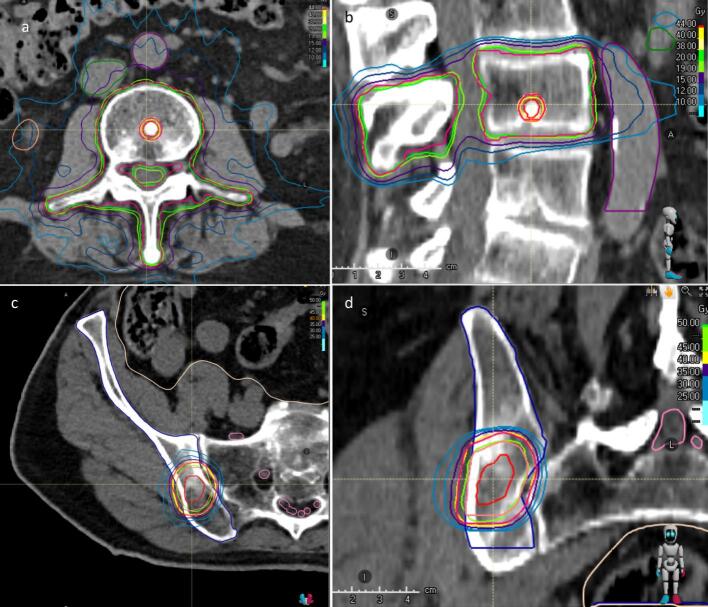
Dose distribution for different SBRT concepts. a) Simultaneous Integrated Boost (SIB) concept for BoM in lumbar spine with 5 × 8 Gy for the boost volume and 5 × 4 Gy for the whole vertebra, and b) GTV plus margin concept for BoM in the right ilium with 5 × 8 Gy prescribed to 80 %. Gross Tumor Volume (GTV) is represented by the red contour, and Planning Target Volume (PTV) is represented by the pink contour. (For interpretation of the references to colour in this figure legend, the reader is referred to the web version of this article.)

A frequently used contouring concept (278 lesions; 81.5 %), especially for non-spine BoM (196; 70.5 %), included the metastases as GTV with only safety margins to PTV (including CTV margins, if applicable) of 0–10 mm (Median 5 mm (95 %CI: 4.8–5.2 mm)) [10]. The GTV for simultaneous integrated boost concept (SIB, 38 lesions; 11.1 %) encompassed the BoM in the metastatically affected vertebral body or bone with a safety margin for the high-dose planning target volume (PTV_SIB_), and the conventional-dose PTV covered the entire affected vertebral body or bone with a small PTV safety margin as described previously [31] (Fig. 1). Thirteen patients (3.8 %), all with spine BoM, received treatment to the metastasis plus adjacent osseous compartments and a small safety margin for the PTV [32].

The dose volume parameters according to ICRU91 for the target volume as well as BED_10_/ BED_4_ and treatment parameters are reported in Table 2, Table 3.

**Table 3:** Dose volume parameters according to ICRU91, presented according to treatment concept.

	SIB-concept	Compartment−/GTV+ margin-concept
Number of treatments	38 (11.1 %) spine: 36/ non-spine: 2	13 (3.8 %, all spine)/ 278 (81.5 %) spine: 82/ non-spine: 196
Mean target volume (GTV) in cc (95 %CI)	2.45 (1.01–3.90)	2.41 (1.47–3.35)
Prescribed dose PTV, median in Gy (95 %CI)	40.0 (37.2–42.8)	30.0 (29.1–30.9)
Number of fractions, median (range)	5 (1–10)	3 (1–10)
Median PTV D_98%_ in Gy (95 %CI)	35.60 (33.23–37.97)	26.02 (25.09–26.96)
Median PTV D_2%_ in Gy (95 %CI)	43.05 (40.27–45.83)	32.24 (31.13–33.35)
Median BED_4_ GTV_mean_ in Gy (95 %CI)	121.40 (114.3–128.41)	107.40 (104.20–110.70)
Median BED_10_ GTV_mean_ in Gy (95 %CI)	76.40 (73.7–79.1)	62.00 (60.20–63.8)
Median BED_4_ PTV D_2%_ in Gy (95 %CI)	130.20 (118.46–141.94)	117.48 (114.11–120.86)
Median BED_10_ PTV D_2%_ in Gy (95 %CI)	80.27 (76.06–84.47)	65.45 (62.96–67.93)

Abbreviations: SIB: Simultaneous Integrated Boost, GTV: Gross Tumor Volume, CI: Confidence Interval, PTV: Planning Target Volume, BED: Biological Effective Dose.

### 3.3 Oncological outcome parameters

**Fig. 2:**
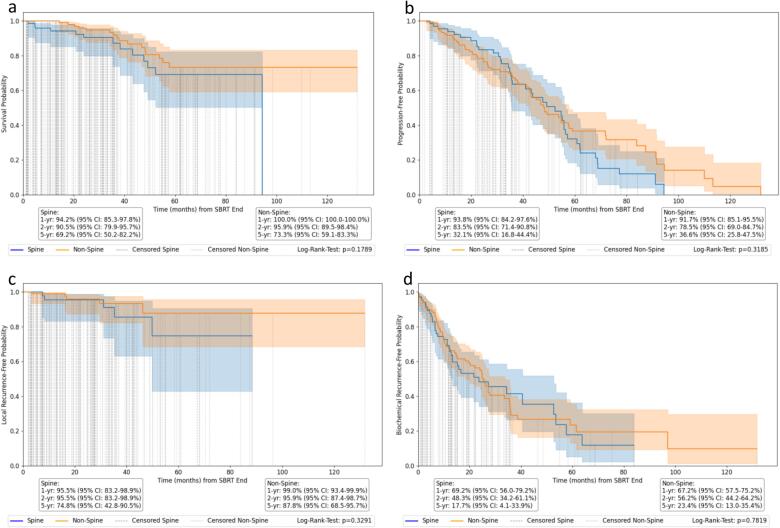
Oncological outcome parameters. Kaplan-Meier curves for a) overall survival (OS), b) progression-free survival (PFS), c) local recurrence-free survival (LRFS), and d) biochemical recurrence-free survival (BRFS) in prostate cancer for spine and non-spine BoM treated with SBRT.

**Fig. 3:**
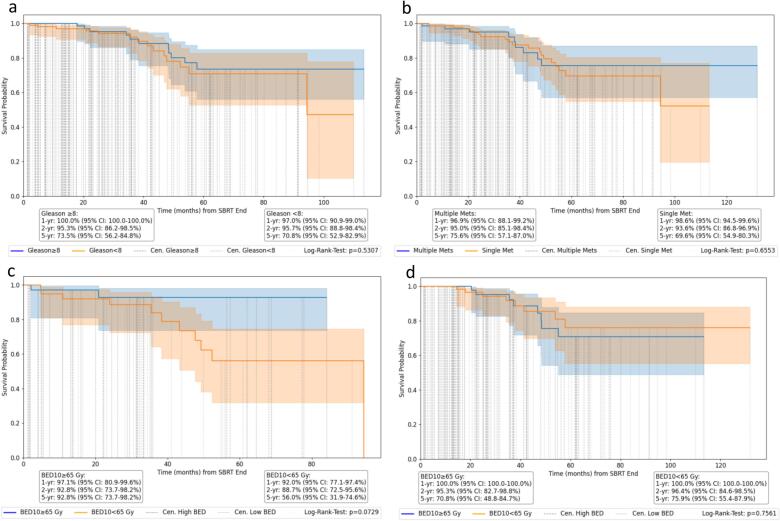
Prognostic variables associated with survival. Kaplan-Meier curves for a) survival probability depending on Gleason score, b) depending on the number on bone metastases (single vs. multiple) in prostate cancer for spine and non-spine BoM treated with SBRT, c) survival probability depending on biologically effective dose (GTV_mean_ BED_10_) for spine, and d) survival probability for non-spine BoM.

Median follow-up time was 28.3 months (95 %CI: 2.0–82.2). The median OS was not reached. 22 patients died during the follow-up period. All analyzed outcome parameters (OS, PFS, LRFS, BRFS) showed no significant difference regarding BoM localization (Fig. 2). For spine BoM, 1-year, 2-year, and 5-year OS rates were 94.2 % (95 %CI: 85.3–97.8 %), 90.5 % (95 %CI: 79.9–95.7 %), and 69.2 % (95 %CI: 50.2–82.2 %), respectively. For non-spine BoM, the corresponding rates were 100.0 %, 95.9 % (95 %CI: 89.5–98.4 %), and 73.3 % (95 %CI: 59.1–83.3 %). Median PFS was 51.1 months for spine BoM and 48.1 months for non-spine BoM. 1-year, 2-year, and 5-year PFS rates for spine BoM were 93.8 % (95 %CI: 84.2–97.6 %), 83.5 % (95 %CI: 71.4–90.8 %), and 32.1 % (95 %CI: 16.8–44.4 %), respectively. For non-spine BoM, the corresponding PFS rates were 91.7 % (95 %CI: 85.1–95.5 %), 78.5 % (95 %CI: 69.0–84.7 %), and 36.6 % (95 %CI: 25.8–47.5 %). 1-year and 2-year LRFS were 95.5 % and 95.5 % (95 %CI: 83.2–98.9 %) for spine BoM, and 99.0 % (95 %CI: 93.4–99.9 %) and 95.9 % (95 %CI: 87.4–98.7 %) for non-spine BoM, respectively. 3.8 % of BoM treated with SBRT recurred locally after a median time of 29.6 months (95 %CI: 18.8–40.3). The BRFS at 1, 2, and 5 years was 69.2 % (95 %CI: 56.0–79.2 %), 48.3 % (95 %CI: 34.2–61.1 %), and 17.7 % (95 %CI: 4.1–33.9 %) for spine BoM, and 67.2 % (95 %CI: 57.5–75.2 %), 56.2 % (95 %CI: 44.2–64.2 %), and 23.4 % (95 %CI: 13.0–35.4 %) for non-spine BoM, respectively. While a trend towards better survival probability for BED_10_ ≥ 65 Gy for spine BoM could be seen (p = 0.07), there was no significant dose dependency for non-spine BoM using BED_10_ (Fig. 3) or for spine and non-spine BoM using BED_4_ (Supplementary Fig. 2). When patients were dichotomized according to their initial Gleason scores between <8 (low/intermediate risk) and ≥8 (high risk), there was no significant difference (p = 0.53) (Fig. 3).

### 3.4 Prognostic variables associated with survival

In multivariate analysis, higher age and application of cancer-directed systemic therapy (ADT or cytotoxic chemotherapy) were associated with worse OS. Elderly patients (HR: 1.09 (95 %CI: 1.04–1.14, p < 0.005)) as well as patients receiving concomitant or sequentially systemic therapy (HR: 2.17 (95 %CI: 0.99–4.75, p = 0.05)) had a higher hazard of death. Other investigated factors such as BoM localization, BED_4_ GTV_mean_ or GTV volume, had no significant impact. Older age and larger GTV volume were significant variables for adverse PFS with a HR of 1.03 (95 %CI: 1.00–1.06, p = 0.04) and 1.03 (95 %CI: 1.00–1.05, p = 0.04), respectively. BoM localization, BED_4_, GTV_mean_, and systemic therapy did not influence PFS significantly. For BRFS, larger BED_4_ for mean dose delivered to the GTV was a significant variable with a HR of 0.99 (95 %CI: 0.98–1.0, p < 0.01), while all other tested parameters had no significant impact (Supplementary Table 1). Due to the low number of local recurrences (3.8 %), a reliable statistical analysis of the correlation between BED_4 or 10_ and LRFS was not possible.

### 3.5 SBRT-related toxicities

The overall rate of acute and late toxicities was low (22.1 %), comprising mostly grade 1–2 events. Only 4.4 % grade 3 toxicities were reported: 2 fractures with need for intervention, 12 out of 341 lesions (3.5 %) presenting with post-treatment pain and 1 deterioration of ureteral obstruction for a pre-existing ureteral dilatation, not necessarily related to the SBRT. No grade 4 or 5 toxicities were observed. The reported fracture rate related to SBRT, without tumor progression, was 0.3 % for acute (one spine lesion without need for intervention) and 1.2 % for delayed fractures (three spine fractures, one of them requiring intervention and one non-spine lesion requiring intervention, Table 4).

**Table 4:** SBRT-related acute and chronic toxicities, reported according to Common Terminology Criteria for Adverse Events (CTCAE) version 5.0 [28] with n = number and additionally relative values in percentage of total number of lesions n = 341 (100 %).

Acute toxicities					
CTCAE Term	Grade 1n (%)	Grade 2n (%)	Grade 3n (%)	Grade 4n (%)	Grade 5n (%)
Fatigue	31 (9.1)	−	−	−	−
Pain	−	10 (2.9)	12 (3.5)	−	−
Dysphagia	2 (0.6)	−	−	−	−
Pathological fracture	1 (0.3)			−	−
Paresthesia	2 (0.6)	−	−	−	−
Dysuria	1 (0.3)	1 (0.3)	−	−	−
Radiodermatitis	2 (0.6)	−	−	−	−
Flatulence	2 (0.6)	−	−	−	−
Nausea	1 (0.3)	−	−	−	−
Incontinence	1 (0.3)	−	−	−	−
Ureter obstruction	−	−	1 (0.3)	−	−
Pneumonitis	−	3 (0.9)	−	−	−
Not specified	1 (0.3)	−	−	−	−

**Chronic toxicities**					

	**Grade 1n (%)**	**Grade 2n (%)**	**Grade 3n (%)**	**Grade 4n (%)**	**Grade 5n (%)**

Pathological fracture	2 (0.6)	−	2 (0.6)	−	−

Abbreviations: CTCAE: Common Terminology Criteria for Adverse Events.

## 4 Discussion

This multicenter study investigated how patients with OMPC respond to SBRT for BoM, especially with regard to patterns of care, efficacy and safety. In the initial SABR-COMET trial, an OS and PFS benefit was reported for MDT added to standard of care for patients with OMD, including more than one third BoM [9]. Most studies on OMD, however, evaluate multiple histologies and/or multiple sites of metastasis in a single assessment [11,[33], [34], [35]]. To achieve reliable results for one of the most common metastatic sites of OMPC, our large real-world cohort focused only on one primary diagnosis with a single metastatic site for spine and non-spine BoM. Our findings support the idea that SBRT is an effective treatment for patients with OMPC. Notably, this can be seen in both spine and non-spine lesions without significant differences in oncologic outcomes. While short-term PFS and OS rates were high, PFS declined steeply at 5 years and BRFS declined even more rapidly for both spine and non-spine BoM. No clear dose dependency was observed regarding survival probabilities irrespective of the BoM localization.

The high effectiveness of bone SBRT was accompanied by low overall toxicity. Aside from mild post-SBRT fatigue, we only observed three grade 3 events other than pain and no acute or late toxicities of higher grades. Especially acute and delayed bone fractures were very low following spine and non-spine bone SBRT. Older age and the need for systemic therapy were associated with worse OS, and older age and larger treatment volumes corresponded with reduced PFS.

A recent systematic review found a pooled 2-year and 4-year OS rate of 90.6 % and 80.1 %, while the pooled 2-year and 4-year PFS rates were 52.7 % and 28.4 %, respectively [14]. In a single institution retrospective study, 87 % of all patients had local control after MDT with a median follow-up time of 18 months [13].

In our cohort, we also found 1- and 2-year LRFS rates above 95 %. These excellent outcome rates may be related to the histology of the primary prostate cancer as also reported in another multicenter retrospective analysis [36]. However, local recurrence might be underreported, as patients with OMPC typically do not receive routine imaging in the absence of a PSA increase in follow-up. One could assume, high LRFS may delay tumor spreading, potentially prolonging the oligometastatic phase, maybe even without further therapies such as ADT. However, nearly two thirds of lesions in our cohort were treated in the context of ongoing or sequential ADT.

In contrast to the reported literature, our 2-year PFS and BRFS rates were even better with 83.5 % and 48.3 % for spine BoM, and 78.5 % and 56.2 % for non-spine BoM, respectively. Nevertheless, PFS and BRFS showed a steep decline after 5 years. This underlines the risk of further metastatic spread in OMPC patients and highlights a potential limitation of SBRT for OMPC [21]. Our results add to the growing evidence that treatment of metastatic lesions with SBRT prolongs PFS compared to observation in patients with OPMC [22,37,38].

The concomitant application of systemic therapy was associated with worse OS (HR: 2.17 (95 %CI: 0.99–4.75, p = 0.05)), suggesting a potential bias that those patients may not have presented with actual OMPC. The recently published RADIOSA trial in patients with OMPC (lymph node metastases and/or BoM) reported a significant longer median clinical PFS if ADT over 6 months is added to SBRT of oligometastases (15.1 months (95 %CI: 12.4–22.8) vs. 32.2 months (95 %CI: 22.4-not reached)) [24]. Similarly, another randomized controlled trial found a significant advantage in PFS for the combination of MDT with ADT compared to ADT alone (HR 0.25 (95 %CI: 0.12–0.55; p < 0.001)) [23]. However, the combination and timing of SBRT and additional systemic therapies for patients with OMPC requires further investigations [[39], [40], [41]].

We saw no significant difference for outcome parameters based on BoM localization. Previous studies such as the *meta*-analysis by Cao et al. [42] have shown the use of higher SBRT doses for non-spine and spine BoM to improve local control. The ESTRO clinical practice guideline for spine SBRT recommends a prescription dose higher than the equivalent of 1x18 Gy (BED_10_ = 50 Gy_10_) [16]. We can now add a trend for higher survival probability for spine BoM in OMPC when a BED_10_ ≥ 65 Gy is applied, although there was no significant difference. Due to the low number of local recurrences (3.8 %) in our trial, a reliable statistical analysis of the correlation between BED_4 or 10_ and LRFS was not possible. However, our results suggest that using higher doses might also be particularly important for better survival probability in spine metastases of OMPC, even though irradiation can be challenging in this area due to adjacent structures [43].

Data on outcome parameters of SBRT to BoM in patients with OMPC stratified by risk classification is limited. In general, previous trials indicate worse PFS or LRFS in patients with higher Gleason scores [38,44]. In our study, we found no significant difference. In the STOMP and ORIOLE trials, the largest benefit of MDT regarding PFS was observed in patients with a high-risk mutation, although PFS was 13.4 months in those without a high-risk mutation, compared with 7.5 months in those with a high-risk mutation [21]. Further studies are needed for stratification of patients who will benefit most from SBRT in the oligometastatic setting [45].

The observed low toxicities in our dataset support the notion that SBRT is well tolerated.

Nearly two thirds of lesions in our cohort were treated with concomitant or sequential ADT, and additional toxicity due to SBRT remained very low [[46], [47], [48]]. Our study documented a very low fracture risk as compared to a previous large *meta*-analysis that pooled data of spine metastases of different primaries and observed a total vertebral fracture rate of 9 % with 1.7 % of lesions requiring surgical stabilization [49]. Chan et al. recently reported a 1-/2-year vertebral fracture rate of 8.4 %/12.3 % for spine SBRT of BoM (different primary sites) with multivariate analysis indicating higher risk of fracture with greater biologically equivalent dose, baseline fracture, and increasing age [50]. The difference might be attributable to the large proportion of osteoblastic lesions in our cohort, which are less susceptible to fractures [51]. Other possible reasons could be the distinct hypofractionation with comparably high number of fractions (median 5) as well as the median patient age of 72 years, which appears to be relatively young compared to the common age (≥86 years) of rising fracture rates [50].

Due to its retrospective study design the current study has some limitations, such as a selection bias. We relied on chart review and clinician reports, so there is potential for under-reporting of events or information that could no longer be traced back. The number of different centers increases the number of cases and thus the informative value of the study, but it also leads to more variability in SBRT protocols and treatment techniques and probably in follow-up procedures of the different institutions. Because of these possible limitations we chose only 5 different university departments with treatment concepts as similar as possible. Nevertheless, there were differences in terms of treatment technique such as linear accelerator or robotic radiosurgery, patient positioning, dose prescription and definition of margins. Furthermore, the reliance on radiological reports for noticing local recurrence and the variability in imaging frequency could have influenced our results. Since there was no control group without SBRT, no clear statement is possible how much SBRT improves survival compared to systemic therapy alone.

## 5 Conclusion

In our large real-world cohort, SBRT of BoM due to OMPC achieved excellent outcomes with low toxicity and particularly low fracture rates in this retrospective multicenter trial. There was no significant difference in outcome parameters between spine and non-spine BoM, while increasing age, need for additional systemic therapy and larger metastatic volume were associated with worse outcomes. In the future, prospective trials will help to further identify the patients benefitting most from this approach, to establish standardized SBRT target volume and dose concepts, as well as standardized follow-up imaging procedures, and to better define the role of concomitant systemic treatments.

## CRediT authorship contribution statement

**Franziska Nägler:** Writing – review & editing, Writing – original draft, Project administration, Methodology, Investigation, Formal analysis, Data curation, Conceptualization. **Isabell Seiler:** Writing – review & editing, Writing – original draft, Resources, Methodology, Investigation, Formal analysis, Data curation. **Sebastian Schäfer:** Writing – review & editing, Visualization, Validation, Software, Resources, Formal analysis, Data curation. **Johannes Meents:** Writing – review & editing, Investigation. **Fabian Lohaus:** Writing – review & editing, Investigation. **Arne Grün:** Writing – review & editing, Investigation. **Olaf Wittenstein:** Writing – review & editing, Investigation. **Kenneth Klischies:** Writing – review & editing, Investigation. **Julia Remmele:** Writing – review & editing, Visualization, Formal analysis, Data curation. **Alexander Rühle:** Writing – review & editing. **Miriam Eckl:** Writing – review & editing, Investigation. **Oliver Blanck:** Writing – review & editing. **Judit Boda-Heggemann:** Writing – review & editing, Supervision, Methodology, Data curation, Conceptualization. **Frank A. Giordano:** Writing – review & editing. **Christos Moustakis:** Writing – review & editing. **Nils H. Nicolay:** Writing – review & editing, Supervision, Resources, Methodology, Conceptualization. **Lena Kästner:** Writing – review & editing, Writing – original draft, Validation, Methodology, Data curation, Conceptualization.

## Ethics approval

This study was reviewed and approved by the local Ethics Committee (127/24-ek). Informed consent to participate in the study was not required. All methods used in this study were carried out in accordance with relevant guidelines and regulations.

## Funding

Supported by the Open Access Publishing Fund of Leipzig University.

## Declaration of competing interest

The authors declare the following financial interests/personal relationships which may be considered as potential competing interests: Alexander Ruehle reports a relationship with Novocure Inc that includes: funding grants, speaking and lecture fees, and travel reimbursement. Alexander Ruehle reports a relationship with Johnson & Johnson that includes: consulting or advisory. Alexander Ruehle reports a relationship with Need Inc. that includes: consulting or advisory. Alexander Ruehle reports a relationship with Merck Healthcare Germany GmbH that includes: consulting or advisory. Alexander Ruehle reports a relationship with AstraZeneca that includes: consulting or advisory. Judit Boda-Heggemann reports a relationship with EBAMed SA that includes: speaking and lecture fees. Judit Boda Heggemann reports a relationship with AstraZeneca that includes: speaking and lecture fees. Judit Boda-Heggemann reports a relationship with BMS that includes: speaking and lecture fees. Lena Kaestner reports a relationship with AstraZeneca that includes: speaking and lecture fees. Miriam Eckl reports a relationship with Siemens Healthineers AG that includes: speaking and lecture fees. Oliver Blanck reports a relationship with DEGRO that includes: board membership. Oliver Blanck reports a relationship with DGMP that includes: board membership. Frank Giordano reports a relationship with TME Pharma that includes: equity or stocks. Frank Giordano reports a relationship with VARIAN, Elekta, Carl Zeiss, Oncomangetx, TME Pharma that includes: consulting or advisory, funding grants, and travel reimbursement. Frank Giordano reports a relationship with Zeiss that includes: speaking and lecture fees. If there are other authors, they declare that they have no known competing financial interests or personal relationships that could have appeared to influence the work reported in this paper.

## Data Availability

The dataset generated during the current study is available from the corresponding author on reasonable request.
