# Supplementary material for: Stereotactic body radiotherapy for spine and non-spine bone metastases in prostate carcinoma – a multicenter cohort analysis
Source: J Bone Oncol. 2025 Sep 11;54:100710. doi: 10.1016/j.jbo.2025.100710 (PMC12466248; doi:10.1016/j.jbo.2025.100710)

**Supplementary Data**

**Supplementary Table 1:** Multivariable Cox proportional hazard regression analysis for Overall Survival, Progression-Free Survival, and Biochemical Recurrence-Free Survival in patients with oligometastatic prostate cancer and stereotactic body radiotherapy.

|  | Overall Survival | | Progression‐Free Survival | | Biochemical Recurrence‐Free Survival | |
| --- | --- | --- | --- | --- | --- | --- |
| Characteristic | p | HR (95% CI) | p | HR (95% CI) | P | HR (95% CI) |
| Age | < .005 | 1.09 (1.04-1.14) | .04 | 1.03 (1.00-1.06) | .07 | 1.03 (1.00-1.06) |
| Spine |  | Reference |  | Reference |  | Reference |
| Non-spine | .71 | 0.88 (0.47-1.68) | .58 | 0.89 (0.59-1.35) | .33 | 1.23 (0.81-1.87) |
| BED_4_ GTV_Mean_ | .99 | 1.00 (0.99-1.01) | .30 | 1.00 (1.00-1.01) | .01 | 0.99 (0.98-1.00) |
| GTV volume | .29 | 1.02 (0.98-1.06) | .04 | 1.03 (1.00-1.05) | .17 | 1.02 (0.99-1.04) |
| No systemic therapy |  | Reference |  | Reference |  | Reference |
| Systemic therapy | .05 | 2.17 (0.99-4.75) | .35 | 0.82 (0.54-1.24) | .19 | 0.77 (0.51-1.14) |

**Supplementary Figure 1:** Pre-existing symptoms before stereotactic body radiotherapy of bone metastases in patients with oligometastatic prostate cancer.


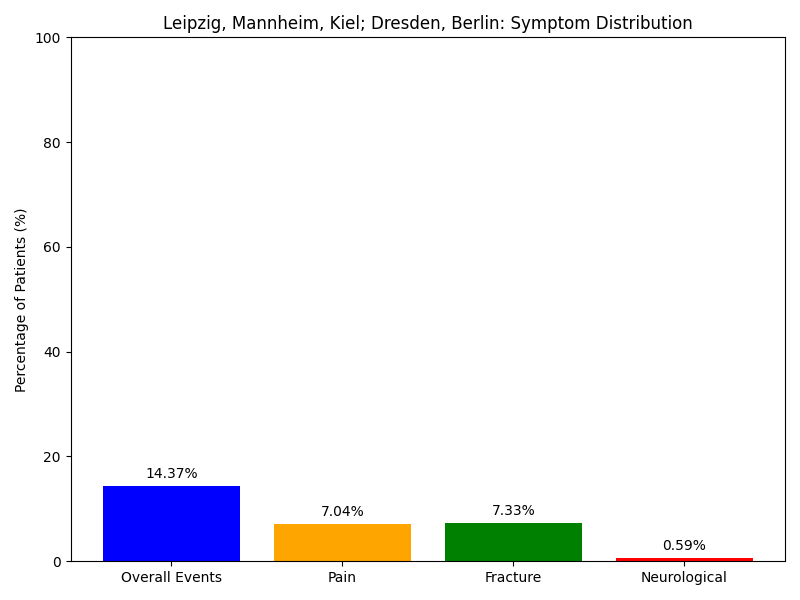


**Supplementary Figure 2:** Kaplan-Meier curves for a) survival probability depending on biologically effective dose (GTV_mean_ BED_4_) for spine and b) non-spine bone metastases and c) survival probability depending on castration resistance for SBRT of bone metastases


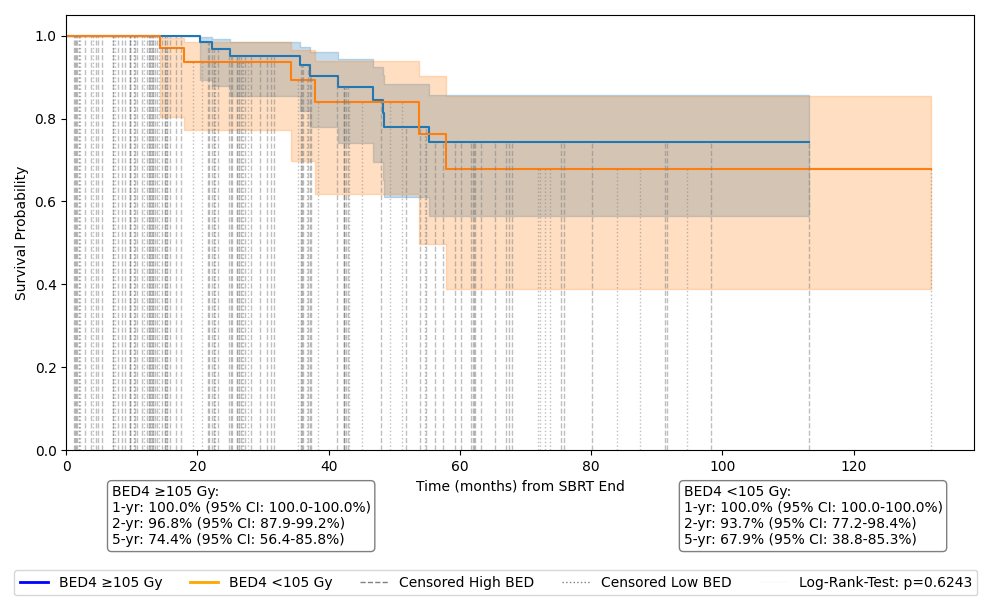

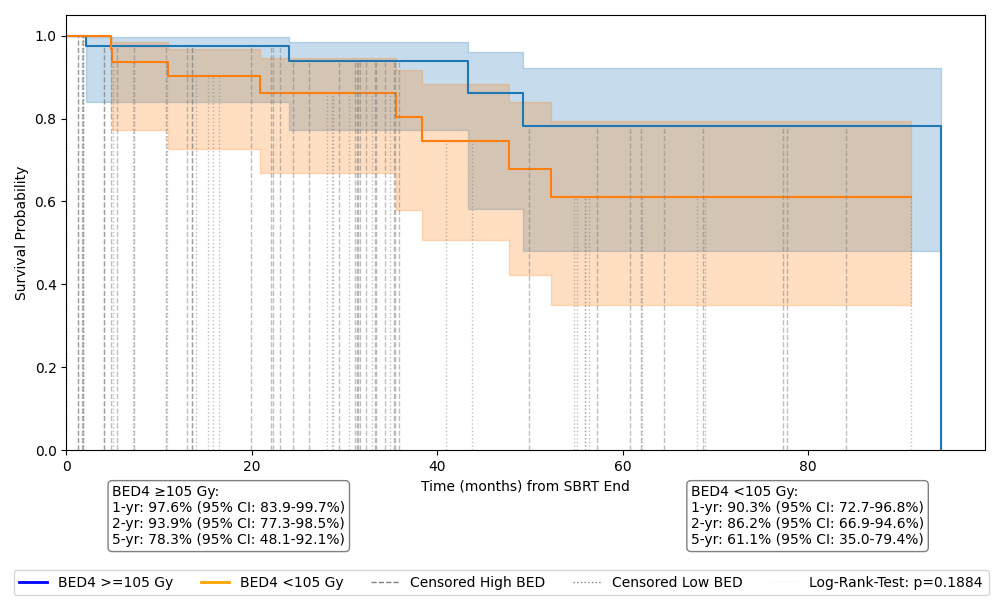


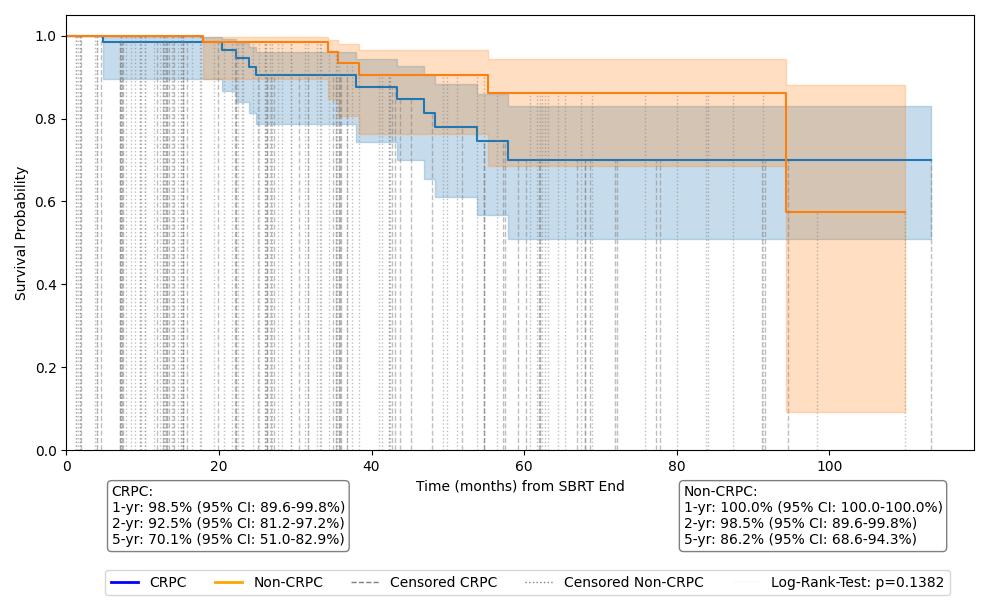

Supplement: Supplementary Data 1 [file mmc1.docx]
